# Prevalence of anemia and its associated factors among rural adolescent girls in West Badewacho district, central Ethiopia: a community based cross-sectional study

**DOI:** 10.3389/fpubh.2025.1567419

**Published:** 2025-06-13

**Authors:** Wondimagegn Kebede, Awoke Girma Hailu, Tadele Hegena, Zeleke Dutamo Agde

**Affiliations:** ^1^School of Public Health, College of Medicine and Health Sciences, Wachemo University, Hossana, Ethiopia; ^2^School of Nursing, College of Medicine and Health Sciences, Wachemo University, Hossana, Ethiopia

**Keywords:** adolescent girls, prevalence of anemia, Badewacho district, rural, central Ethiopia

## Abstract

**Background:**

Anemia is closely interconnected with the five global nutrition targets, including stunting, low birth weight, childhood overweight, exclusive breastfeeding, and wasting. However, previous studies in Ethiopia have predominantly focused on populations other than adolescents or have relied on hospital-based surveys with limited geographical coverage, resulting in limited evidence in this segment of the population. This study therefore aimed to assess the prevalence and determinants of anemia among adolescents in the West Badewacho District, central Ethiopia.

**Methods:**

A community-based cross-sectional study was conducted among 548 adolescent girls in the West Badewacho district, central Ethiopia, in June 2022. A systematic random sampling technique was employed to select the study participants. Data were collected using a pretested structured questionnaire. A 10 microliter (μL) blood sample was collected from each participant. Data entry and analysis were conducted using EpiData version 4.6 and SPSS version 25, respectively. Adjusted odds ratios (AORs) with 95% confidence intervals (CIs) were computed and variables with *p* < 0.05 in the multivariable logistic regression analysis were considered significantly associated with anemia.

**Results:**

The prevalence of anemia was 25.9% (95% CI: 20.7, 30.1). Among the anemic participants, 17.5% had mild anemia (hemoglobin level 10.0–11.9 g/dL) and 8.4% had moderate anemia (hemoglobin level 8.0–9.9 g/dL). The strongest predictors of anemia were household family size of five or more (AOR: 8.02; 95% CI: 3.97, 16.17), menstrual blood flow lasting 5 days or more (AOR: 7.64; 95% CI: 2.02, 28.94), and lack of intestinal parasite treatment or deworming (AOR: 3.94; 95% CI: 1.63, 9.52). Iron-folate intake during adolescence was found to be protective against anemia (AOR: 0.64; 95% CI: 0.11, 0.93).

**Conclusion:**

Anemia among adolescent girls was a moderate public health concern in the study area. The strongest predictors of anemia were larger household family size, prolonged menstrual blood flow, and lack of deworming, while iron-folate supplementation was protective. Efforts to reduce anemia should focus on promoting iron-folate supplementation, addressing prolonged menstrual bleeding, improving treatment for intestinal parasites, and providing targeted interventions for larger families.

## Introduction

Anemia is a condition in which the number and size of red blood cells, or the hemoglobin concentration, falls below an established cut-off value (< 12 g/dL), consequently impairing the capacity of the blood to transport oxygen around the body (1). According to the 2008 global database report of the World Health Organization, anemia is affecting over 1.62 billion individuals (2). Anemia is an indicator of both poor nutrition and poor health (3). Female adolescents are at higher risk for anemia than male adolescents. In Ethiopia, adolescents constitute about 48% of the population, of which about 25% are female (4).

Anemia is associated with the five other global nutrition targets (stunting, low birth weight, childhood overweight, exclusive breastfeeding, and wasting). In particular, the control of anemia in women of child bearing age is essential to prevent low birth weight and perinatal and maternal mortality later in life (3).

The likelihood of iron deficiency anemia resurfaces for both boys and girls during the growth spurt of adolescence. Adolescent girls represent a specifically at-risk demographic, as they have elevated needs for iron and experience significant iron losses from their bodies. Of the total population, adolescent girls form 22% and estimates suggests that about 25–50% of girls become anemic by the time they reach menarche (5). Ethiopian adolescent girls are not exceptions to this problem and for example, according to the Ethiopia Demographic and Health Survey (EDHS) 2016, 18.2% of women aged 15 to 19 years were anemic (6).

Recent aggregated data reveal that the prevalence of anemia in Ethiopian adolescent girls is relatively high at 23.02% (95% CI: 17.21–28.84). Factors such as living in rural areas and being older (ages 15–19) notably elevate the risk, with odds ratios of 2.05 and 2.13, respectively (7). While the burden of anemia among adolescent girls in Ethiopia has progressively declined and the country has made significant strides in reducing nutritional anemia, understanding the specific determinants and the magnitude among this age group in rural community remains limited (8).

Despite national progress in reducing anemia, rural adolescent girls remain an understudied population. Most prior research has focused on urban or institutional settings, limiting understanding of anaemia’s burden and determinants in rural communities where dietary diversity is low and parasitic infections are common (9). Other recent studies also have primarily focused on populations other than adolescents indicating there is a dearth of evidence in this segment of population (3). This study therefore aimed to addresses these gaps by assessing the prevalence and factors associated with Anemia among adolescent girls in West Badewacho District, Central Ethiopia.

## Methods and materials

### Study area and period

The research was conducted in the West Badewacho District in June 2022. The district is part of the Hadiya zone in Central Ethiopia, situated 112 km from Hossana, the central Ethiopian regional state capital city, and 350 km south of Addis Ababa, the capital city of Ethiopia. West Badewacho district is densely populated, with a total population of 118,453, consisting of 55,304 males and 65,149 females, as per the district report of 2021. There are 22 kebeles, the lowest administrative unit in Ethiopia, in the district, along with 4 health centers, 22 health posts, and 11 private clinics providing healthcare services to the population.

### Study design

A community based cross-sectional design was employed to assess the magnitude of anemia and associated factors among adolescent girls.

### Inclusion and exclusion criteria

The study included adolescent girls aged 10–19 years who had been residing in the study area for at least 6 months. However, girls who were pregnant or breastfeeding, those whose parents did not provide consent for blood testing, and those with severe illnesses were excluded.

### Sample size determination and sampling procedure

Sample size was determined using a single population proportion formula by considering the 39% prevalence of anemia from a study conducted in Ambo, West Shewa, Ethiopia (9), confidence level of 95%, 0.05 margin of error, and design effect of 1.5. Finally, a total of 548 adolescent girls participated in the study.

Out of 22 rural kebeles, seven kebeles, namely Elfeta, Sibeya, Ya Bukuna, Wada 01, Sephera, 2nd Kotto, and Danema 01, were selected by simple random sampling. The sampling frame was prepared using the household number from the kebele administration. Then the calculated sample size of 548 was proportionally allocated to each selected kebele based on its size of adolescence in each kebele. Based on the size of the adolescent girls, the sampling interval (K) value was calculated for each kebele. Then, first house hold was selected by lottery method.

### Data collection and measurement

Data was collected using structured questionnaires involving nutritional status (height, weight, BMI) and hemoglobin level with a HemoCue machine (HemoCue® Hb 201 + analyzer) which is widely validated and recommended by the World Health Organization for anemia screening in community-based studies, with high accuracy and reliability comparable to laboratory methods (2). A blood sample of 10 microliters (μL) was collected from each participant. The structured questionnaires were designed to collect data on socio-demographic and economic characteristics, dietary habits, health history, and hemoglobin level. Trained nurses, who had previous experience in data collection, conducted anthropometric measurements, including height, weight, and Body Mass Index (BMI) of the study participants. Height was measured using a stadiometer, weight was measured using a digital weight scale, and BMI was calculated as weight (kg) divided by height squared (m^2^).

Hemoglobin levels were assessed using a HemoCue machine according to the World Health Organization (WHO) guidelines for anemia diagnosis, which used HemoCue® Hb 201 + System (2). A 10 microliter (μL) blood sample was collected from each participant and analyzed using the HemoCue machine to determine their hemoglobin levels. Then the hemoglobin level was classified mild (11–11.9 g/dL), moderate (8–10.9 g/dL), and severe (< 8 g/dL): a girl was considered anemic if her hemoglobin level was < 12 g/dL (Anaemia among adolescent girls in three districts in Ethiopia) (10).

### Data quality control

A pre-test was conducted on 10% of the sample size in kebele, which was not selected for the actual study and necessary amendments were made before the commencement of data collection. Consequently the data collectors and supervisors received two-days training on the study’s objective, method, and contents. Additionally they were trained on how to maintain the confidentiality and privacy of the study participants. English version of the questionnaire was translated in to local language (Hadiyisa) by bilingual experts and then the local versions translated back into English by professionals to check its consistence. Blood sample for Hgb tests was obtained by finger pricking of participants’ using lancet. It was analysed using the portable haemoglobin meter HemoCue. Anemia was then defined according WHO cut off point (haemoglobin level < 12 g/dL). Discarding of the lancet with an open container was made and readings performed immediately. The internal consistency of the questionnaire was assessed, yielding a Cronbach’s alpha of 0.7. Furthermore, regular supervision, spot-checking, and reviewing of the completed questionnaire were employed on daily basis to ensure data quality.

### Data processing and analysis

The data were coded and entered into EpiData version 4.6 and then exported to SPSS version 25 for analysis. Data were summarized and presented using graphs, charts, tables, and figures. We employed a bivariable logistic regression analysis to assess the association between the dependent variable and each independent variable. Variables with *p*-value <0.25 in the bivariable logistic regression analysis had been included in the final model, multivariable logistic regression analysis. Adjusted odds ratios with their 95% CI were computed, and variables having *p* < 0.05 in the multivariable logistic regression model were considered as significantly associated with anemia. A multicollinearity test was done among the independent variables using the variance inflation factor (VIF). Model fitness was assessed with the assumptions of the Hosmer and Lemeshow goodness of fit test.

## Result

### Socio-demographic characteristics

A total of 548 sample participants responded to the interview, making a response rate of 100%. The mean age of the women was 14.95 (SD + 2.48). Four hundred thirty-six (79.6%) were single by their marital status, and three hundred eighty-one (69.5%) of participants were Hadiya in ethnicity. Three hundred fifty-one (64.0%) of participants were Protestant religion followers. Two hundred twenty-nine (41.8%) were completed secondary schools, and one hundred ninety-four (35.5%) of participants’ mothers completed secondary school. One hundred ninety-two (35.0%) of participants had a family size of less than 3. One hundred seventy-three (31.5%) of participants fathers were farmers by occupation. One hundred ninety-four (35.4%) participants’ families had > 3,500 birr as monthly income (Table 1).

**Table 1:** Socio-demographic characteristics of participants at West Badewacho district, central Ethiopia, June, 2022 (*n* = 548).

Variables	Categories	Frequency (n)	Percentage (%)
Marital status	Single	436	79.6
	Married	112	20.4
Ethnicity	Hadiya	381	69.5
	Kembata	19	3.5
	Wolaita	24	4.4
	Amhara	8	1.4
	Others	116	21.2
Religion	Protestant	351	64.0
	Orthodox	114	20.8
	Muslim	83	15.2
Respondent education	Cannot read and write	9	2.1
	Grade 1–4	146	26.6
	Grade 5–8	159	29.0
	Grade 9–12	229	41.8
	College and above	14	2.6
Mother education	No formal	123	22.4
	Primary	165	30.1
	Secondary (grade 9–12)	194	35.5
	College above	66	12.0
Family size	<= 3	192	35.0
	4–5	184	33.6
	>5	172	31.4
Occupation father	Government employee	162	29.7
	Merchant	129	23.5
	Farmer	173	31.5
	Daily labor	84	15.3
Occupation mother	Government employee	113	20.6
	Housewife	189	34.5
	Merchant	246	44.9
Family income in birr per month	<500	51	9.3
	500–1,499	84	15.3
	1,500–2,499	114	20.8
	2,500–3,499	105	19.2
	>3,500	194	35.4

### Dietary-related factors and feeding habits

Regarding the types of food consumed as part of their diet, the majority of participants, 62.9%, included grain foods such as maize, wheat, white roots, and tubers. Additionally, 79.2% of participants consumed green leafy vegetables, while 70.99% of the participants did not consume meat per week for nutrition. More than half (52.2%) of the participants included fruits like oranges, mangoes, and bananas in their diet, and 56.02% consumed dairy products like milk weekly. A majority of participants, 60.1%, also consumed pulses such as beans, peas, lentils, and nuts, while 85.3% did not consume eggs. Furthermore, 57.3% consumed vitamin A-rich vegetables like potatoes and carrots, and 60.7% consumed fats and oils. Additionally, 62.4% drank tea or coffee, with about half (55.2%) consuming it three or more times per week. A smaller percentage (20.2%) included Kocho (local food) as a main part of their diet. The majority, 81.02% of participants, had lunch daily, while 68.4% consumed less than two cups of tea per week. About two-thirds of the participants (74.3%) had three meals per day. Around half (50.18%) of the respondents received nutritional education, and the majority (79.56%) fell within the BMI range of 18.5–24.99. More than half (55.5%) had access to pipe or protected spring water sources, and 86.3% did not use boiled water for consumption. Additionally, 58.2% had a medium diversity score in their diet (Table 2).

**Table 2:** Group of food items consumed to measure individual dietary diversity of adolescent girls at West Badewacho district, Southern Ethiopia, June, 2022 (*n* = 548).

Variables	Categories	Frequency	Percentage (%)
Grains like maize, wheat, white roots, tubers	Yes	345	62.9
	No	203	37.1
Green leafy vegetables eating	Yes	434	79.2
	No	114	20.8
Meat eating per week	Yes	159	29.01
	No	389	70.99
Fruits like (orange, mango, banana) eating	Yes	286	52.2
	No	262	47.8
Milk (dairy) per week	Yes	307	56.02
	No	241	43.98
Pulses (beans, peas, lentils, nuts)	Yes	329	60.1
	No	219	39.9
Eggs	Yes	75	14.7
	No	473	85.3
Vit. A rich vegetables like potato and carrot	Yes	314	57.3
	No	234	42.7
Fats and oils	Yes	215	39.2
	No	333	60.8
Drink tea/coffee	Yes	342	62.4
	No	206	37.6
Frequency meat eaten	< 3 times per week	246	44.8
	> = 3 times per week	302	55.2
Main diet	Maize	109	19.9
	Milk Product	110	20.1
	Teff	109	19.9
	Wheat Bread	109	19.9
	Kocho	111	20.2
Lunch daily	Yes	444	81.02
	No	104	18.98
Number tea/week	Less than 2 cup	375	68.4
	2–4 cups	122	22.3
	Greater than 4 cups	51	9.3
Meal frequency per day	< 3 times	24	4.3
	3 times	417	74.3
	4–5 times	120	21.4
Nutritional education	Yes	275	50.18
	No	273	49.82
BMI	17–18.499	83	15.15
BMI	16.1–16.99	29	5.29
BMI	18.5–24.99	436	79.56
Drinking water	Pipe/protected spring	304	55.5
	Well/spring/rain water	244	44.5
Boil water	Yes	75	13.7
	No	473	86.3
DDS	Low	53	9.6
	High	176	32.2
	Medium	319	58.2

DDS, Dietary Diversity Score; BMI, Body Mass Index.

### Physiological factors

From the total participants, the majority, 88.9%, had begun their menstrual cycle, and 80.5% of participants had menstrual blood flow duration of 3–5 days. More than half (54.74%) of the respondents had a menstrual cycle every 21–35 days, and 71.7% had a regular menstrual cycle. The majorities, 86.1% of respondents, avoided food during menstruation, and 86.1% of adolescents had no pain during menstruation. Moreover, 74.6% of the participants agree with iron tablet intake during adolescence. Out of the total participants, 79.4% had been treated for malaria, whereas 77.0% had not been treated for intestinal parasites (Table 3).

**Table 3:** Physiological (menstrual) related factors of adolescent girls at West Badewacho district, central Ethiopia, June, 2022 (*n* = 548).

Variables	Categories	Frequency	Percentage (%)
Menstruation began	Yes	487	88.9
	No	61	11.1
Menstrual blood flow duration	1–2 days	62	11.3
	3–5 days	441	80.5
	More than 5 days	45	8.2
Menstrual cycle	<21 days	132	24.09
	21–35 days	300	54.74
	> = 35 days	116	21.17
Menstrual cycle regularity	Regular	393	71.7
	Irregular	155	28.3
Foods avoided during menstruation	Yes	76	13.9
	No	472	86.1
Illness	Tonsillitis	22	4.1
	Stomach pain	33	6.0
	Renal infection	21	3.8
	Normal (no)	472	86.1
Agree with iron tablet intake during adolescence	Yes	409	74.6
	No	139	25.4
Malarial treatment	Yes	435	79.4
	No	113	20.6
Intestinal parasite treatment	Yes	126	23.0
	No	422	77.0
Abortion history	Yes	48	8.7
	No	500	91.3

### The prevalence of anemia

**Figure 1:**
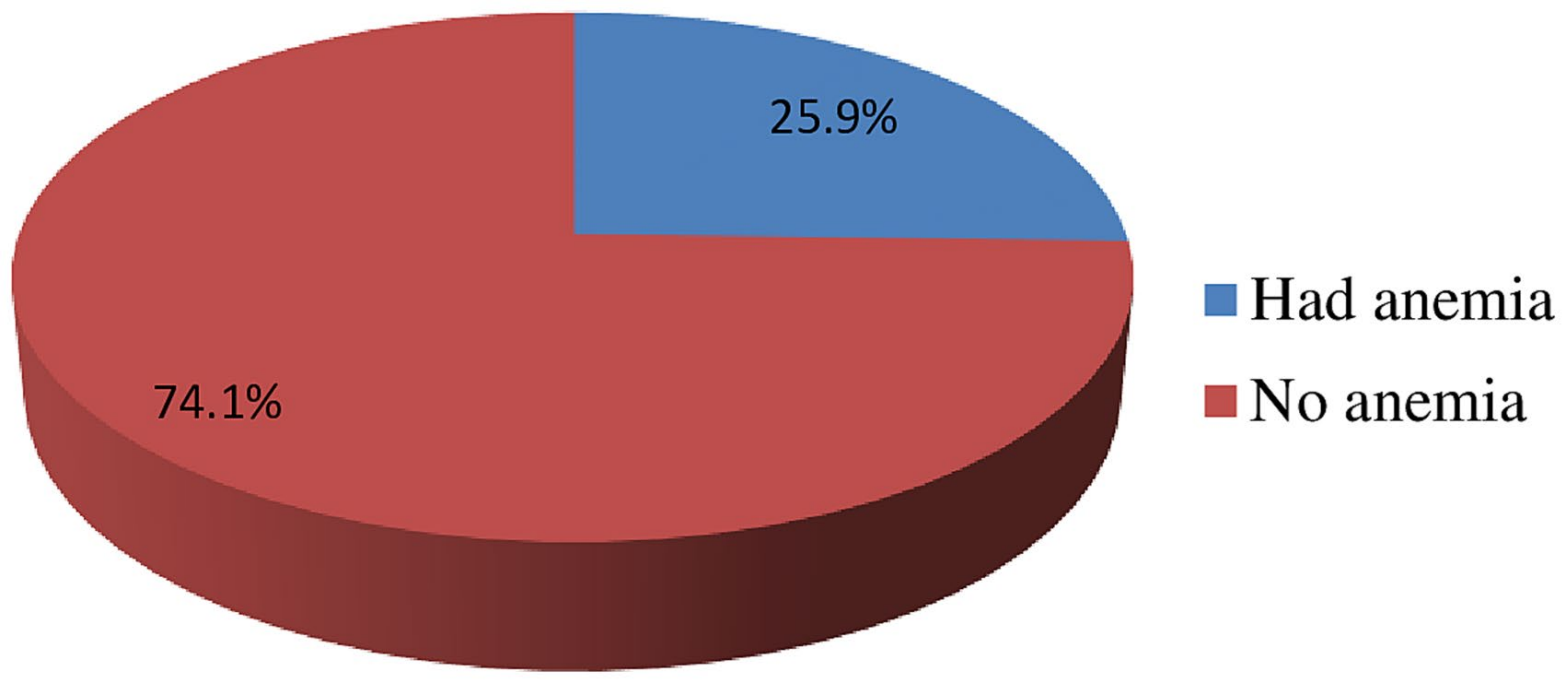
Prevalence of anemia among rural adolescent girls categorized as mild anemia (hemoglobin 10–11.99 g/dL) and moderate anemia (hemoglobin 8–9.99 g/dL), based on WHO classification at West Badewacho district, central Ethiopia, June, 2022 Ethiopia (*n* = 548).

In this study, the prevalence of anemia was 25.9% (95% CI: 20.7, 30.1) among adolescent girls. Out of this, 17.5% of them had mild anemia (hemoglobin level of 10–11.99 g/dL), and the remaining 8.4% of the participants had moderate anemia (hemoglobin level of 8–9.99 g/dL) (Figure 1).

### Factors associated with iron deficiency anemia

In the final multivariable logistic regression analysis, iron-folate intake, menstrual bleeding ≥5 days, household size, and lack of deworming were significantly associated with iron deficiency anemia among adolescent girls. Accordingly, menstrual blood flow for > = 5 days was one of the significantly associated factors with the prevalence of iron deficiency anemia among adolescent girls. Adolescent girls who faced menstrual blood flow for > = 5 days during their menstrual cycle were nearly 8 times more likely to be anemic than their counterparts (AOR: 7.64; 95% CI: 2.02, 28.94). Adolescent girls who was agree to use iron tablet during adolescent were 36% less likely being anemic compared with those who did not agree to use iron tablet during adolescent (AOR: 0.64;95% CI: 0.11, 0.93). Moreover, adolescent girls whose house-hold family size 5 and above were almost 8 times at higher risk of iron deficiency anemia when compared to those whose house-hold family size less than 5 (AOR: 8.02; 95% CI: 3.97, 16.17). Furthermore, adolescent girls who had no history of intestinal parasite treatment or deworming were almost 4 times more likely being anemic than those who were treated/ dewormed for intestinal parasite in the last 6 months (AOR = 3.94; 95% CI: 1.63, 9.52) (Table 4).

**Table 4:** Factors associated with iron deficiency anemia among rural adolescent girls at West Badewacho district, central Ethiopia, June, 2022 (*n* = 548).

Variables	Category	Anemia		COR (95% CI)	AOR (95% CI)
		Yes	No		
Iron foliate intake during adolescent	Yes	125	284	0.31 (0.13, 0.86)**	0.64 (0.11, 0.93)**
	No	17	122	1.0	1.0
Menstrual blood flow	1–2 days	28	34	1.0	1.0
	3–5 days	81	360	0.51 (0.14, 1.92)	0.79 (0.26, 2.42)
	> = 5 days	33	12	7.92 (1.59, 39.54)***	7.64 (2.02, 28.94)***
Family size	<=3	33	159	1.0	1.0
	<= 4	61	123	0.25 (0.01–2.52)	0.25(0.0153–2.5)
	> = 5	48	124	6.51 (3.78, 11.19)**	8.02 (3.97, 16.17)***
Abortion history	Yes	32	16	0.14 (0.01–0.82)	0.13 (0.11, 1.19)
	No	110	390	1.0	1.0
Malaria illness	Yes	121	314	0.59 (0.18, 0.95)	1.86 (0.69, 4.97)
	No	21	92	1.0	1.0
Occupation of mother	Government employee	27	86	1.0	1.0
	Merchant	79	167	1.40 (0.67, 2.92)	0.56 (0.11, 2.81)
	Housewife	36	153	2.41 (1.15, 5.02)*	0.81 (0.17, 3.85)
Occupation of father	Government employee	58	104	1.0	1.0
	Merchant	43	86	2.19 (1.04, 4.58)*	2.31 (0.84, 6.35)
	Farmer	32	141	2.26 (1.14, 4.48)*	2.51 (0.98, 6.47)
	Daily labour	9	75	2.63 (1.11, 6.22)*	3.16 (0.96, 10.40)
IPs treatment	Yes	17	109	2.69 (1.21 4.86)**	3.94(1.63, 9.52)**
	No	125	297	1.0	1.0

COR, Crude Odds Ratio; AOR, Adjusted Odds Ratio; IPs, Intestinal parasites; 1.0, Reference category; ***p < 0.001, **p < 0.01, *p < 0.05.

## Discussion

This study aimed to determine the prevalence of anemia and its factors affecting rural adolescent girls in the West Badewacho district. The study revealed the prevalence of anemia among these girls was relatively high when compared to previous studies, with an overall rate of 25.9% (95% CI: 20.7, 30.1). This finding aligns with a similar study in Dembia District, Northwest Ethiopia (25.5%) (11) and Jimma (26.7%) (12). However, it was lower than findings from Ghana (56.5%) (13). The possible reasons for this variation might be due to difference in socioeconomic status, study settings, and study period. However, this is higher than findings from Bonga, southwest Ethiopia study (15.2%). This may be due to irregular, prolonged and heavy amount of menstruation, high intestinal parasite infections and undernutrition, poor knowledge on nutrition and anemia, and skipping of meals (14). The relatively high prevalence of anemia among rural adolescent girls in West Badewacho district underscores the urgent need for targeted interventions, such as iron and folic acid supplementation programs and community-based nutritional education to improve awareness about balanced diets and the prevention of anemia.

The study found that prolonged menstrual bleeding, iron folate intake, household family size, and history of intestinal parasite treatment were significantly associated with anemia among adolescent girls. Prolonged menstrual bleeding for more than 5 days increased iron deficiency anemia among adolescent girls by almost 8-fold. Similar findings were reported from the studies conducted in Yemen (15), Bahir-Dar (16), and Jimma, Ethiopia (12, 17). This is might be due to more losses of iron as a result of menstrual bleeding. This might be due to more losses of iron as a result of menstrual bleeding. This condition lowers blood hemoglobin concentrations and hence causes anemia.

Adolescent girls who were from family sizes of 5 and above were almost 8 times higher risk of being anemic when compared to those who were from family sizes of four or less, which is nearly in line with the findings from Bahir Dar city, Ethiopia (16). This might be because, as family size increases, food in sufficient quantities might not be achieved consistently for all individuals. And also all individuals within those households might not get sufficient resources, like financial resources, to obtain appropriate foods for a nutritious diet. Moreover, adolescent girls who used iron tablets during menstruation were 36 percent less likely to be anemic than those who did not use iron tablets during their adolescence. This might be because, at these stages of life (adolescence), pregnancy, and childhood, the requirement of iron is high. As the need for iron increases during these times of growth and development, and in combination with household food access problems, it leads to anemia.

Furthermore, adolescent girls who were not treated/dewormed for intestinal parasite were almost 4 fold being anemic than those who were treated/dewormed for intestinal parasite. This finding aligns with results from studies conducted in Bahir Dar City, Ethiopia (16), and India (18). A possible reason for this variation might be due to parasite infections such as hookworms, ascaris, and schistosomiasis consuming red blood cells. All these conditions lower blood hemoglobin concentrations and hence result in anemia. The findings imply the need for strengthening deworming programs and routine intestinal parasite treatment as part of adolescent health services to reduce the burden of anemia.

The following recommendations provided for respective stakeholders based on our findings scaling up school-and community-based iron and folic acid supplementation programs, as endorsed by Ethiopia’s National Nutrition Programme II. Integrating routine deworming and nutrition education into adolescent health services. Leveraging community health workers and peer education models to improve awareness and compliance with anemia prevention strategies. Addressing household food insecurity through multispectral approaches involving agriculture, education, and water/sanitation improvements.

One limitation of this study was the inability to establish a cause-and-effect relationship between the outcome and explanatory variables due to the cross-sectional nature of the study design. Another limitation of the study was the inability to evaluate local cultural influences on dietary habits and health practices that could impact the prevalence of anemia.

## Conclusion

The findings of this study demonstrate that anemia among adolescent girls in the study area represents a moderate public health concern. Factors such as iron folate intake, prolonged menstrual bleeding of 5 days or more, being a member of a larger family size, and lack of treatment for intestinal parasites were identified as significant contributors to iron deficiency anemia among these girls. Efforts to reduce anemia among adolescent girls should focus on promoting iron-folate supplementation, addressing prolonged menstrual bleeding, improving treatment for intestinal parasites, and providing targeted interventions for larger families. The following recommendations also provided for respective stakeholders based on our findings scaling up school-and community-based iron and folic acid supplementation programs, as endorsed by Ethiopia’s National Nutrition Programme II. Integrating routine deworming and nutrition education into adolescent health services. Leveraging community health workers and peer education models to improve awareness and compliance with anemia prevention strategies. Addressing household food insecurity through multispectral approaches involving agriculture, education, and water/sanitation improvements. Future research should include longitudinal studies to examine the long-term effects of anemia on reproductive health and overall development in adolescent girls, which will provide deeper insights for designing effective interventions.

## Data Availability

The raw data supporting the conclusions of this article will be made available by the authors, without undue reservation.
